# Isolated Pancreatic Hypoplasia: A Rare but Significant Radiological Finding

**DOI:** 10.4103/1319-3767.56090

**Published:** 2009-10

**Authors:** Rajul Rastogi, Rakesh Kumar, Sumeet Bhargava, Vaibhav Rastogi

**Affiliations:** Yash Diagnostic Center, Yash Hospital and Research Center, Civil Lines, Kanth Road, Moradabad - 244 001, UP, India

Pancreatic hypoplasia refers to underdevelopment of pancreatic parenchyma which arises from either the ventral or dorsal anlage. Pancreatic hypoplasia secondary to agenesis of the dorsal pancreas is a rare congenital anomaly, with less than 20 cases reported till date.[1,2] Though the majority of these patients present with abdominal pain (which is either nonspecific or typical of pancreatitis) or diabetes mellitus, the disorder may rarely remain quiescent and be detected only incidentally.[2] Agenesis of the dorsal pancreas is usually associated with various anomalies, such as polysplenia syndrome, wandering spleen, interruption of the inferior vena cava, hemiazygos and azygos continuation, symmetrical liver, anomalous hepatic fissure or lobe, left-sided inferior vena cava, median gall bladder, inverted gallbladder and stomach, and intestinal malrotation; there may also be a combination of multiple visceral anomalies.[3] In this article, the authors report a rare case of hypoplasia of the pancreas that was detected incidentally on imaging; there was no other coexisting anomaly or complication. The case is reported because of its rarity. The emphasis is on the differential diagnosis, coexisting anomalies, and complications.

A 45-year-old alcoholic, nondiabetic male patient with clinical hepatomegaly and an unremarkable past medical history came for ultrasonography of the abdomen. Laboratory tests, including blood glucose levels, HbAlc levels, liver function tests, and kidney function test were within normal limits.

Ultrasonography revealed mild hepatomegaly with grade I fatty infiltration. The pancreas body and tail were not optimally visualized; however, the head of the pancreas appeared normal. The main pancreatic duct was not dilated.

**Figure 1:**
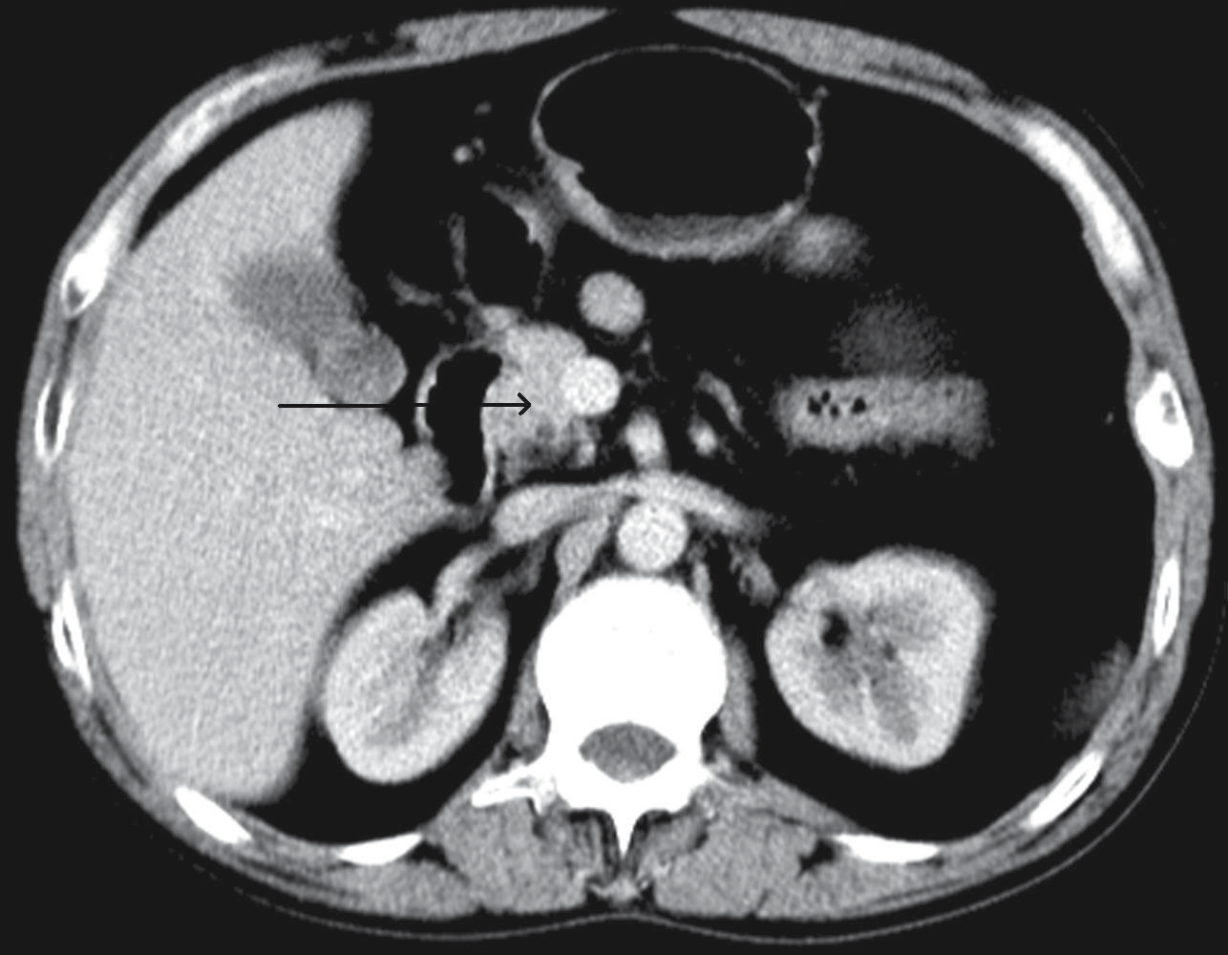
Axial contrast-enhanced CT image shows absence of neck, body, and tail of the pancreas; there is a truncated head (black arrow)

**Figure 2:**
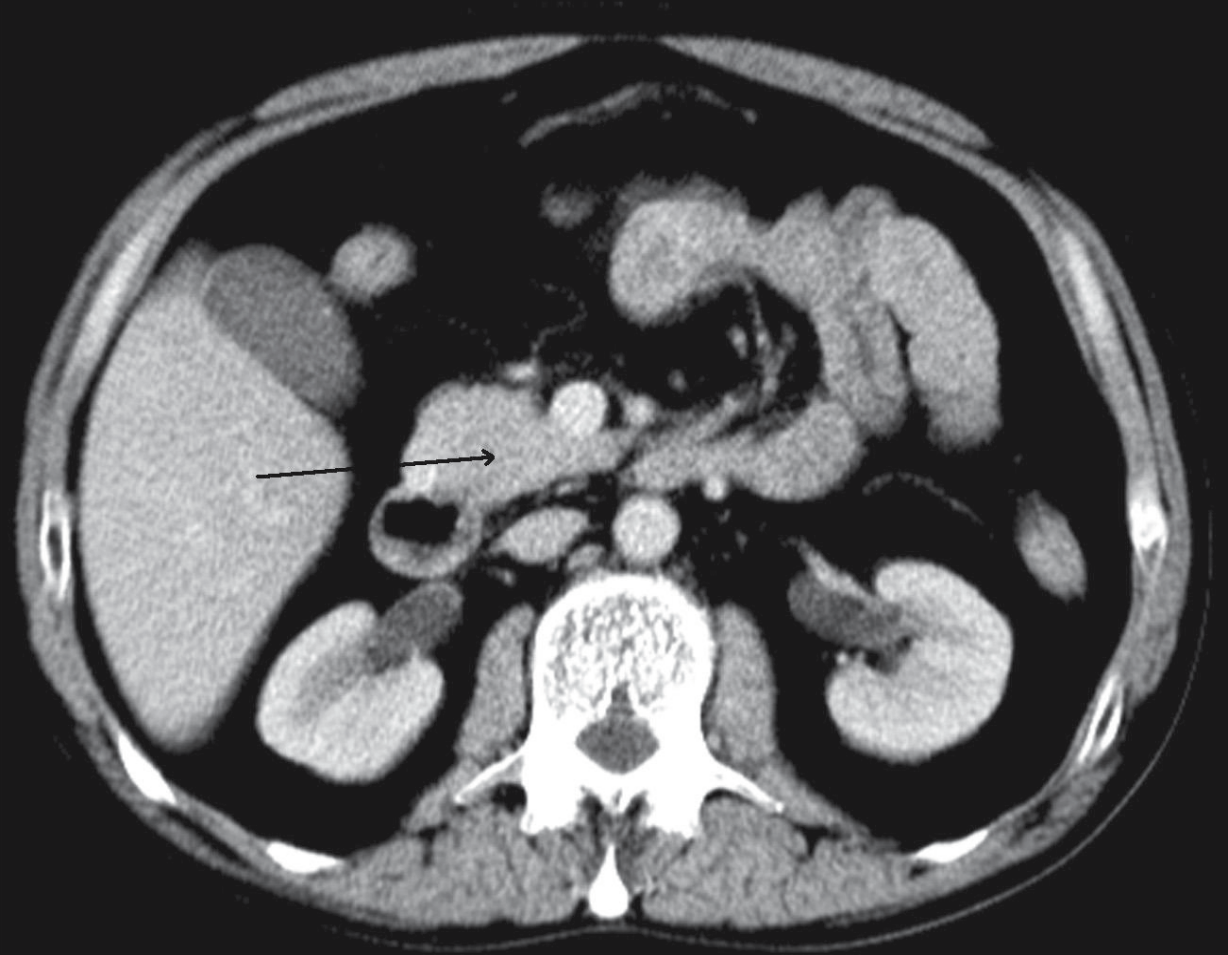
Axial contrast-enhanced CT image shows a normalappearing truncated head with a normal-appearing uncinate process (black arrow)

Contrast-enhanced computed tomography (CT) of the abdomen revealed complete absence of the neck, body, and tail of the pancreas [Figure 1]. The head of the pancreas, though normal in attenuation and pattern of enhancement, appeared flattened and truncated at the anterosuperior part [Figure 2]. Mild hepatomegaly was also noted. The other intra-abdominal structures, including the spleen, gall bladder, and inferior vena cava appeared unremarkable.

Based on the clinical and CT findings, we arrived at the diagnosis of isolated, uncomplicated, agenesis of the dorsal pancreas or hypoplasia of the pancreas secondary to agenesis of the dorsal pancreas.

**Figure 3:**
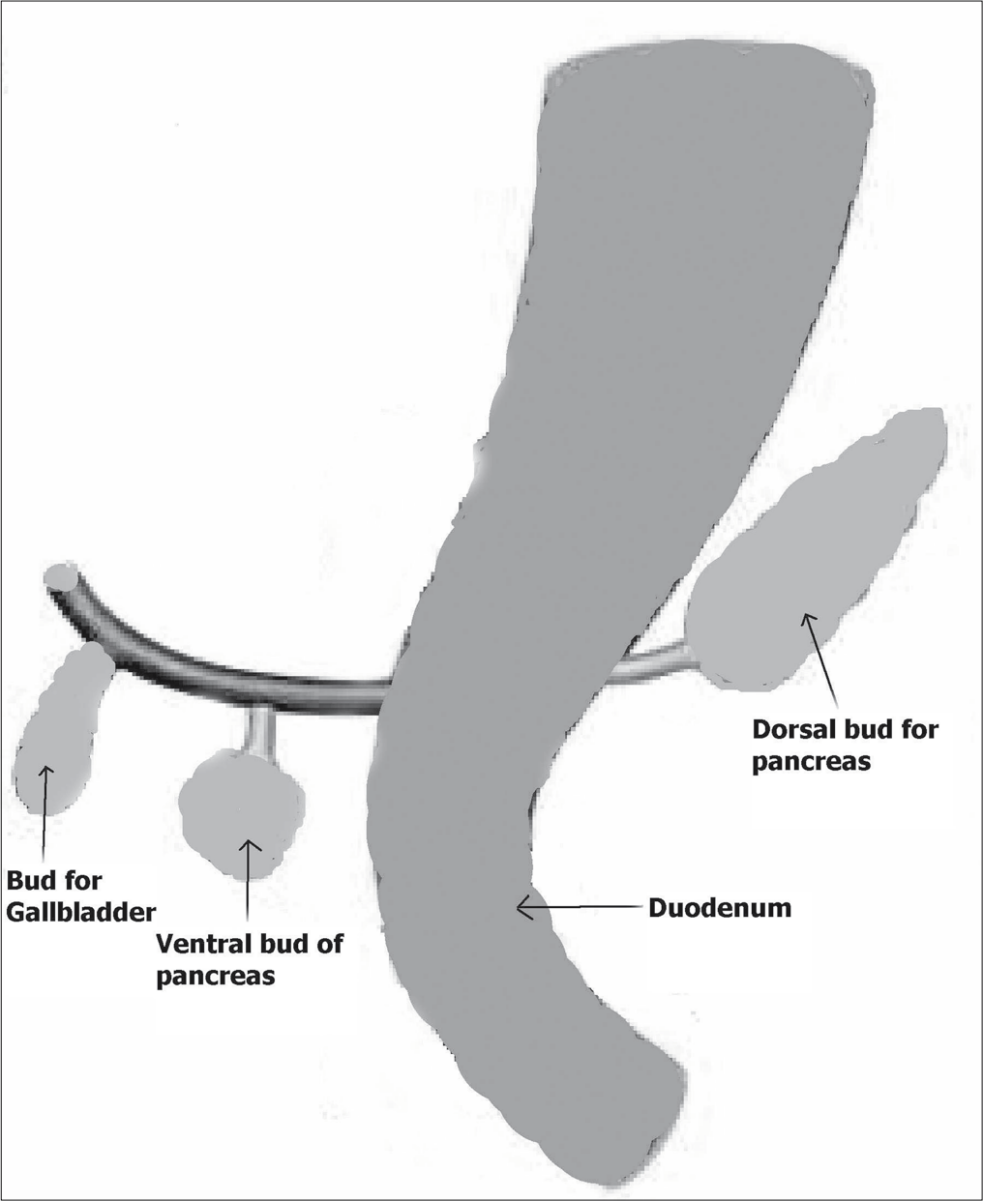
Schematic figure shows embryonic development of pancreas from the ventral and dorsal buds arising from either side of the duodenum

Pancreatic hypoplasia refers to congenital underdevelopment of pancreas and is often referred to as partial agenesis of the pancreas; this agenesis can involve the ventral or dorsal anlage. Figure 3 shows the ventral and dorsal buds from which the head; and neck, body and tail of the pancreas, respectively, develop. When the dorsal bud fails to develop, agenesis of the dorsal pancreas results. Agenesis of the dorsal pancreas is more common than ventral agenesis. Complete agenesis of the pancreas is extremely rare and is incompatible with life.[1] Severe hypoplasia of the pancreas can be associated with mutations involving the HNF1β gene.

The common clinical presentation is abdominal pain, which may be nonspecific or secondary to pancreatitis. Recurrent pancreatitis is quite common.[2] Many patients present with diabetes mellitus.[4] Sometimes, the patient may present with steatorrhea or other signs of exocrine insufficiency.[5]

Imaging in a case of agenesis of the dorsal pancreas reveals a short and truncated head of pancreas, with absence of the neck, body, and tail of the organ. Based on endoscopic retrograde cholangiopancreatography (ERCP), absence of the dorsal anlage is categorized as complete (when the duct of Santorini and the minor duodenal papilla are absent) and partial (when they are remnant).[1] ERCP and magnetic resonance cholangiopancreatography will show a short ductal system in the ventral pancreas, with absence of any ductal system in the body and tail region.[5]

Important differential diagnoses are carcinoma of head of pancreas with secondary atrophy of the distal body and tail of pancreas, pancreatic lipomatosis (fatty replacement of pancreatic parenchyma), and pancreatic divisum. All can be differentiated easily by imaging. Treatment is not required in asymptomatic patients.[5]
